# The Effectiveness of Near-Field Communication Integrated with a Mobile Electronic Medical Record System: Emergency Department Simulation Study

**DOI:** 10.2196/11187

**Published:** 2018-09-21

**Authors:** Kwang Yul Jung, Taerim Kim, Jaegon Jung, JeanHyoung Lee, Jong Soo Choi, Kang Mira, Dong Kyung Chang, Won Chul Cha

**Affiliations:** 1 Department of Emergency Medicine Samsung Medical Center Sungkyunkwan University School of Medicine Seoul Republic Of Korea; 2 Department of Computer Engineering Seoul Digital University Seoul Republic Of Korea; 3 Department of Information Strategy Samsung Medical Center Sungkyunkwan University School of Medicine Seoul Republic Of Korea; 4 Department of Digital Health Samsung Advanced Institute for Health Sciences & Technology Sungkyunkwan University Seoul Republic Of Korea

**Keywords:** near-field communication, electronic medical records, emergency department, mobile health, mHealth

## Abstract

**Background:**

Improved medical practice efficiency has been demonstrated by physicians using mobile device (mobile phones, tablets) electronic medical record (EMR) systems. However, the quantitative effects of these systems have not been adequately measured.

**Objective:**

This study aimed to determine the effectiveness of near-field communication (NFC) integrated with a mobile EMR system regarding physician turnaround time in a hospital emergency department (ED).

**Methods:**

A simulation study was performed in a hospital ED. Twenty-five physicians working in the ED participated in 2 scenarios, using either a mobile device or personal computer (PC). Scenario A involved randomly locating designated patients in the ED. Scenario B consisted of accessing laboratory results of an ED patient at the bedside. After completing the scenarios, participants responded to 10 questions that were scored using a system usability scale (SUS). The primary metric was the turnaround time for each scenario. The secondary metric was the usability of the system, graded by the study participants.

**Results:**

Locating patients from the ED entrance took a mean of 93.0 seconds (SD 34.4) using the mobile scenario. In contrast, it only required a mean of 57.3 seconds (SD 10.5) using the PC scenario (*P*<.001). Searching for laboratory results of the patients at the bedside required a mean of only 25.2 seconds (SD 5.3) with the mobile scenario, and a mean of 61.5 seconds (SD 11.6) using the PC scenario (*P*<.001). Sensitivity analysis comparing only the time for login and accessing the relevant information also determined mobile devices to be significantly faster. The mean SUS score of NFC-mobile EMR was 71.90 points.

**Conclusions:**

NFC integrated with mobile EMR provided for a more efficient physician practice with good usability.

## Introduction

### Background

An emergency department (ED) is often characterized by chaos and inefficiency [1]. It is where the severity of a patient’s injury or distress varies and changes unpredictably. The location of patients also changes based on their clinical process or test results, which may become available at different times. Physicians need to check changed locations and laboratory results frequently by walking back and forth between personal computer (PC) stations and patients’ beds. In a fast-paced ED, such interruptions cause physicians to waste a substantial amount of time and ultimately result in patient dissatisfaction [2]. As the installation of PCs to all bedsides is costly and ineffective regarding space utilization, better alternatives must be considered.

### Related Technologies

The ED providers strive to improve the efficiency of the workflow by exploiting advanced technologies. With the emergence of electronic medical records (EMR), mobile EMR systems are receiving increasing attention as mobile devices (ie, mobile phones, tablets), and mobile apps are becoming more common [3,4]. The portability and near ubiquity of mobile EMR allow health care providers to access patient records wherever they are needed [5].

Near-field communication (NFC) is widely used in various communication apps. In the field of health care, usage scenarios including patient identification [6], blood transfusion [7], drug administration [8], medical staff tracking [9], and medical record access [10] have already been proven. The wave of NFC technology in the health care field has been combined with the internet of things technologies [11]. Through a combination of mobile EMR systems, NFC technology can improve workflow using bedside technology [12].

### Study Objectives

Numerous studies have investigated the qualitative and quantitative benefits of each technology separately [12-14]. However, the efficacy of the combined technologies for improved physician productivity in health care has not been investigated to date. More rigorous quantitative studies investigating usability estimates are required to develop and eventually adopt such systems in practice. Additionally, it is essential to determine a system’s effectiveness in clinical settings. This study aims to determine the effectiveness of an NFC-integrated mobile EMR system regarding physician turnaround time in an ED.

## Methods

### Study Setting

This simulation study took place in an academic ED in Seoul, South Korea. The study was reviewed and approved by the Samsung Medical Center Institutional Review Board (IRB no. SMC 2018-01-144-001).

The ED is part of a tertiary academic teaching hospital with approximately 9000 daily outpatients and 2000 inpatient beds [15]. The ED holds 69 treating beds. The number of annual visits is approximately 79,000. Although the ED is heavily equipped with PCs at each station (84 PCs total), there are no PCs at the bedsides. Most of the beds are not in private rooms but are open to stations except isolation beds.

**Figure 1:**
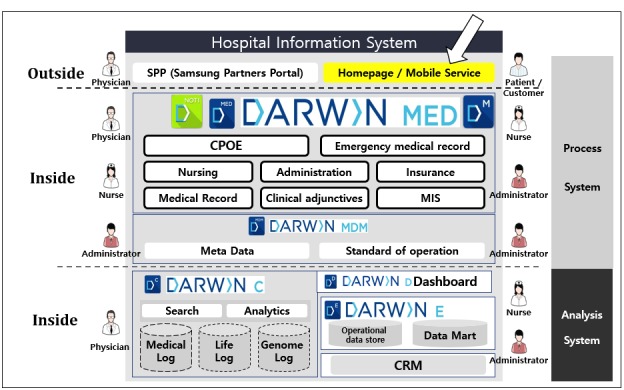
Overall schematic description of the hospital information system architecture relationship at the Samsung Medical Center. DARWIN: data analytics and research window for integrated knowledge; CPOE: computerized physician order entry; MIS: management information system; MDM: master data management; CRM: customer relationship management.

The hospital developed the proposed mobile EMR system. It operates on the institution’s EMR system, which was also developed internally. The overall system had a significant update in July 2016. Figure 1 shows a schematic of the architecture of the EMR system.

**Figure 2:**
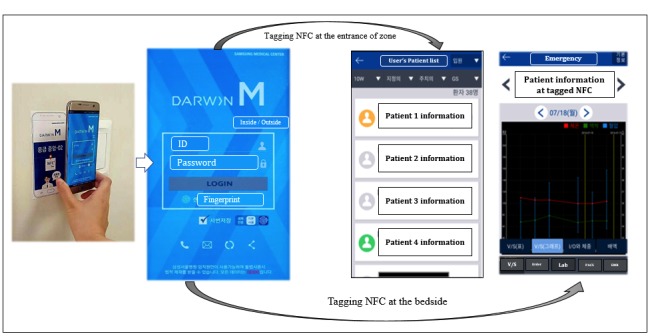
Usage scene when the mobile electronic medical records (EMR) communicate with the near-field communication (NFC) system and the display of the mobile EMR progression after tagging NFC. V/S: vital sign.

The mobile EMR uses an Android app that gives physicians access to inpatient, outpatient, and emergency department information. Users can log into the system with their fingerprint and search for locations, clinical notes, vital signs, laboratory results, and medical images. The NFC function was implemented in April 2017. When a physician links the EMR mobile device to an NFC tag which contains information about its location, the mobile app is automatically initiated. Using fingerprint authentication, physicians can log in and view the EMR of patients at the location that corresponds with their NFC location. When tagging NFC tags at the entrance of a specific zone, the list of patients at the tagged NFC zone who are in charge of mobile device users is popped up. Figure 2 shows the access process.

### Study Participants

Physicians who worked in the ED during the study were asked to participate. Physician participants were recruited between April 1 to April 20, 2018. Among the 35 ED physicians, 25 (71%) agreed to participate in this study.

### Study Scenarios and Sensitivity Analysis

**Figure 3:**
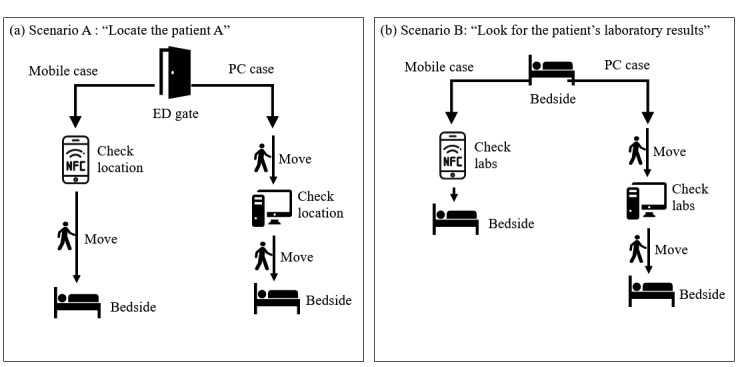
Schematic view of simulation scenarios. (a) Locating the patient. (b) Looking up laboratory results for the patient. ED: emergency department; PC: personal computer.

**Figure 4:**
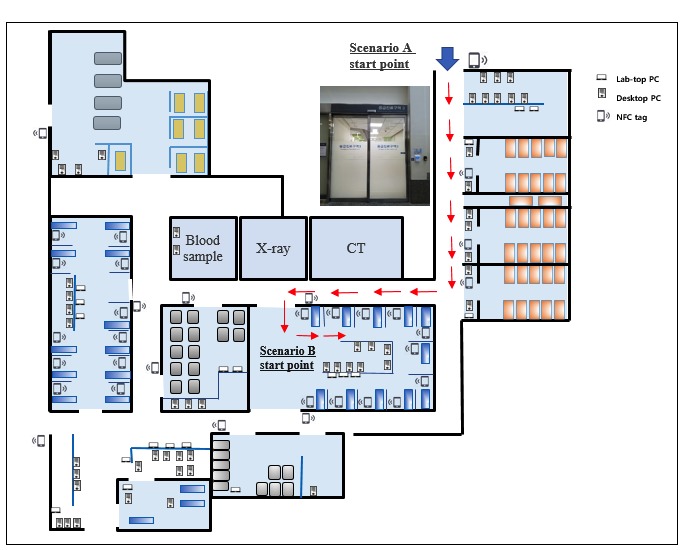
The flow of locating the patient and the scenario place in the emergency department. CT: computed tomography; NFC: near-field communication; PC: personal computer.

After a brief introduction, the physicians went through 2 sequential scenarios. The first scenario (scenario A) involved locating patients in the ED from the ED gate. Physicians were given the name of a patient and were required to locate them using either a PC EMR at the nearest site in the ED gate or mobile EMR. After locating the patient, the participant was guided to reach their bedside. The second scenario (scenario B) involved looking up a laboratory result from the bedside. Physicians were brought to a patient’s bedside and were required to determine a specific laboratory result using either a mobile device or PC interface. As there were no PCs at the bedside, physicians had to perform a few steps to identify available PCs and return with a report. The steps in each scenario are shown in Figure 3, and the flow of each scenario is shown in Figure 4.

Physicians were randomly assigned to follow either scenario A or B using either a mobile device (mobile case) or a PC (PC case) as described in Multimedia Appendix 1. An independent observer recorded the activities with a camera and completed a case report form with time stamps during the process. Patients were not simulated. Real patients were accessed in the emergency department. However, since we used only partial patient data such as name, location, and laboratory data which was already available, the clinical condition did not influence the study’s outcome.

We performed a sensitivity analysis using the data without considering movement intervals. This test was performed to determine whether or not there was a consistent outcome if the condition allowed for more available PCs, which are at the gate and the bedside.

### Survey

After completing all scenarios, physician participants responded to 10 questions using the system usability scale (SUS). The SUS is composed of a 5-point Likert scale rated from 1 (strongly disagree) to 5 (strongly agree) that investigates the usability of the NFC-integrated mobile EMR system [16]. The SUS score calculation formula is as follows:

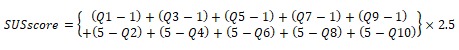



### Measurement and Outcome

The primary metric was the length of turnaround time for each scenario. The secondary metric was the usability of the system, as graded by the study physician participants. We collected demographic data from each participant and recorded the time intervals of each step of the process for both scenarios. We also analyzed time intervals among groups sorted by age, gender, and occupation. Afterward, the SUS questionnaires were collected and analyzed.

### Statistical Analysis

Continuous variables are expressed in terms of mean and standard deviations (SD), whereas categorical variables are expressed in frequencies and percentages. The time mean difference was examined using a paired *t*-test. A value of *P*<.05 was considered to be statistically significant. As descriptive statistics could not confirm a normal distribution of participants between the 2 dependent groups divided by age, gender, and occupation, the Mann-Whitney U test was applied for time interval difference analysis.

## Results

### Main Outcome

Among 25 physician participants, 14 (56%) were male, and 11 (44%) were female. The general characteristics of the participants are shown in Table 1.

**Figure 5:**
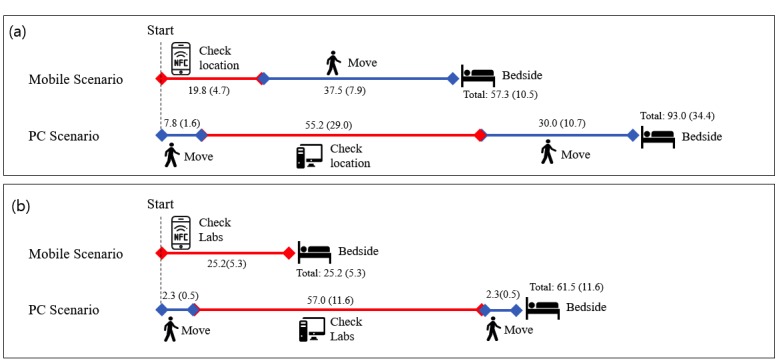
Graphical view of main results. (a) Locating the patient. (b) Looking up laboratory results for the patient. NFC: near-field communication; PC: personal computer.

It required a mean of 93.0 seconds (SD 34.4) to locate the patient from the entrance of the ED in the PC case but only a mean of 57.3 seconds (SD 10.5) in the mobile case, which was significantly faster (*P*<.001). Accessing laboratory results at the patient’s bedside required a mean of only 25.2 seconds (SD 5.3) in the mobile case compared to a mean of 61.5 seconds (SD 11.6) in the PC case. These data were statistically significant (*P*<.001). A schematic comparison is shown in Figure 5.

### Sensitivity Analysis

We compared the time required for login with the time for finding relevant information. Login using the mobile device EMR required a mean of 13.1 seconds (SD 2.9) for scenario A and a mean of 12.5 seconds (SD 2.1) for scenario B. Login by PC took longer with a mean of 36.2 seconds (SD 15.2) for scenario A and a mean of 30.5 seconds (SD 7.7) for scenario B. The differences in time were statistically significant (*P*<.001). Finding the location of patients after login required a mean of only 6.8 seconds (SD 3.6) using the mobile device, whereas it took a mean of 18.9 seconds (SD 16.9) using a PC. Accessing a specific laboratory test result required a mean of 12.8 seconds (SD 5.3) using the mobile device and a mean of 26.5 seconds (SD 8.0) using a PC. These data were statistically significant (*P*<.001). The results are shown in Table 2.

### Survey

The mean SUS score of NFC-mobile EMR was 71.90 points. The results are shown in Table 3.

**Table 1:** Characteristics of the physician participants.

Participant characteristic		Value	
Age (years), mean (SD)		30.6 (4.9)	
**Age groups (years), n (%)**			
	≥30		12 (48)
	<30		13 (52)
**Gender, n (%)**			
	Male		14 (56)
	Female		11 (44)
**Occupation, n (%)**			
	Intern		4 (16)
	Resident		15 (60)
	Specialist		6 (24)
Time worked at hospital (years), mean (SD)		4.6 (4.0)	

**Table 2:** A comparison between the 2 scenarios of time spent on specific tasks.

Task			Time (seconds), mean (SD)			*P* value
			Mobile case		Personal computer case	
**Scenario A**						
	Login	13.1 (2.9)		36.2 (15.2)		<.001
	Accessing relevant information	6.8 (3.6)		18.9 (16.9)		<.001
	Total	19.8 (4.7)		55.2 (29.0)		<.001
**Scenario B**						
	Login	12.5 (2.1)		30.5 (7.7)		<.001
	Accessing relevant information	12.8 (5.3)		26.5 (8.0)		<.001
	Total	25.2 (5.3)		57.0 (11.6)		<.001

**Table 3:** Score results (n=25) from the system usability scale (SUS) to assess the near-field communication mobile emergency medical record (NFC-mobile EMR).

Question	Mean (SD)
1. I think that I would like to use this NFC-mobile EMR frequently.	3.92 (0.95)
2. I found the NFC-mobile EMR unnecessarily complex.	1.76 (0.83)
3. I thought the NFC-mobile EMR was easy to use.	4.40 (0.50)
4. I think that I would need the support of a technical person to be able to use the NFC-mobile EMR.	2.72 (1.10)
5. I found that the various functions in the NFC-mobile EMR were well-integrated.	4.24 (0.72)
6. I thought there was too much inconsistency in the NFC-mobile EMR.	4.20 (0.64)
7. I would imagine that most people would learn to use the NFC-mobile EMR very quickly.	4.48 (0.59)
8. I found the NFC-mobile EMR very cumbersome to use.	1.56 (0.51)
9. I felt very confident using the NFC-mobile EMR.	3.88 (0.78)
10. I needed to learn a lot of things before I could get going with the NFC-mobile EMR.	1.92 (0.57)
Total score	71.90 (7.61)

## Discussion

### Principal Findings

This study aimed to improve physician efficiency by reducing the time spent walking to check patient information with the aid of the technological integration between NFC and mobile device EMR. To the best of our knowledge, this is the first study to examine the efficiency of this system and comparing it with the PC EMR. The mobile total turnaround time for performing tasks was significantly reduced in both scenarios. Sensitivity analysis showed that mobile device EMR incorporated with NFC was significantly faster than PC-integrated EMR regarding login time and accessing laboratory results.

As the familiarity of mobile device use could be different among the demographic groups, we compared the total time interval difference between PC and mobile cases. Multimedia Appendix 2 shows that the mobile case was consistently faster for all groups. However, there were significant differences in the time interval between age and occupation during scenario B. These findings are contrary to the general belief that the younger generation is more familiar with newer technology [17]. A further study on mobile device familiarity is needed because the simulation was done with a small sample size.

We also evaluated usability with the SUS questionnaire. The SUS was used after the physician participant had an opportunity to use the system being evaluated. A score over 70 on the questionnaire (range 0-100) indicated that the NFC-integrated mobile device EMR was “acceptable,” and the adjective rating was “good” [18]. There was no significant statistical difference among groups based on age, gender, and occupation.

### Advantages and Disadvantages

Various measures have been implemented to address ED inadequacies. Improving the ED work efficiency is one crucial in-hospital factor. Ideal physical structures for work have already been demonstrated [19]. Several studies have shown the positive effect of developing clinical guidelines and protocols for effective evaluation of efficiency [20,21]. Newer technologies such as radio frequency identification-integrated point-of-care testing [22], triage kiosks [23], and dashboards [24] have been well studied. Ubiquitous near patient access to EMR via NFC is determined to be useful in this regard. Compared to installing new structures in an already heavily equipped ED, implementing an NFC tag system is a relatively easy way to improve workflow regarding cost and space utilization.

As most mobile EMR functions are more readily accessible with PCs, our study paid attention to superiority only available in mobile EMR. Mobile device systems outperform PC systems concerning mobility and personalization (at the provider level, and patient level). We have measured the turnaround time as the primary outcome of these merits. Thus, we have shown that physicians can gain access to information without physically moving the location of their patients.

Portability of mobile EMR could be improved by incorporating accessibility through NFC. Our study revealed a statistically significant difference in login time which was more effective by mobile EMR than by PC EMR (Table 2). A previous study by Holden [25] demonstrated that the issue of accessibility to EMR such as system login and system response time could negatively impact the usability of mobile EMR. NFC integrated response and fingerprint login at the location of interest using a mobile device could be beneficial because the process is simplified and less time-consuming. This system also appears to reduce security concerns from failed logouts or departure without logging out, by using the individual’s mobile device.

An increase in the length of time physicians spend at the bedside is likely to increase patient satisfaction [26]. With this bedside technology, the physician can show radiologic results or laboratory results to patients who cannot ambulate.

Inconsistent loading time due to varying network coverage could be a disadvantage for this technology. For example, mobile devices without NFC function cannot be used. Physicians might routinely tend to use PC EMR because PC EMR covers mobile EMR. A previous study by Duhm et al [14] demonstrated that a physician usually underestimates actual time savings during their professional capacity. The results of this study make a compelling argument and provide preliminary evidence in support of adequately addressing this tendency, particularly concerning reduced workflow using mobile EMR with NFC functionality.

However, to enhance emergency physician performance, a multidimensional approach is required, rather than a single tool. ED processes are complicated, with multiple steps from various providers often originating from outside the ED.

### Limitations

First among the limitations of this study is that this investigation was conducted at a single center. Additional studies conducted at multiple centers or EDs are needed to improve the generalizability of our conclusions.

Secondly, participants had different levels of familiarity with mobile devices and NFC tags. Only some participants were familiar with NFC because the system was built over a year ago, which might cause bias.

Thirdly, each participant encountered various encumbrances because this study was conducted in an actual emergency room. For example, when attempting to locate a patient in the middle of a scenario, the nearest PC may have been occupied by another staff member, which led to the physician being forced to use a PC that was further away. Also, while moving to a patient’s bedside, there was an occasion when a participant was forced to stop because a moving stretcher cart or medical staff member blocked the aisle. In addition, some of the PCs used were comparatively slow. As mentioned above, unpredictable circumstances might influence the overall time measured for each scenario. As shown in Multimedia Appendix 3, the variability of turnaround time fluctuated. However important, these events could not be systemically quantified.

Finally, the usability assessment for NFC-mobile EMR via SUS could be overrated because responses were filled out immediately after performing scenarios, which in most cases, resulted in the superiority of NFC-mobile EMR. Further studies could investigate usability over a more extended period of the physician’s working practice.

### Conclusion

NFC-integrated mobile EMR is effective for reducing the turnaround time of physicians when practicing in the field and has excellent usability.

## Appendix

Multimedia Appendix 1 Randomly allocated scenario quest for each participant. PC: personal computer.

Multimedia Appendix 2 Comparison of time spent among age, gender, and occupation for each scenario.

Multimedia Appendix 3 Graphical comparison between mobile and personal computer scenario time of all participants.

## Abbreviations

ED: emergency department
EMR: emergency medical record
NFC: near-field communication
PC: personal computer
SUS: system usability scale
