# Supplementary material for: The Effectiveness of Near-Field Communication Integrated with a Mobile Electronic Medical Record System: Emergency Department Simulation Study
Source: JMIR Mhealth Uhealth. 2018 Sep 21;6(9):e11187. doi: 10.2196/11187 (PMC6231820; doi:10.2196/11187)
Supplement: Multimedia Appendix 1 [file mhealth_v6i9e11187_app1.pdf]

Multimedia Appendix 1.

Participant no.	Simulation 1	Simulation 2	Simulation 3	Simulation 4
1	Scenario A mobile case	Scenario A PC case	Scenario B mobile case	Scenario B PC case
2	Scenario B PC case	Scenario B mobile case	Scenario A PC case	Scenario A mobile case
3	Scenario A mobile case	Scenario A PC case	Scenario B mobile case	Scenario B PC case
4	Scenario B PC case	Scenario B mobile case	Scenario A mobile case	Scenario A PC case
5	Scenario B mobile case	Scenario B PC case	Scenario A mobile case	Scenario A PC case
6	Scenario A PC case	Scenario A mobile case	Scenario B mobile case	Scenario B PC case
7	Scenario A PC case	Scenario A mobile case	Scenario B PC case	Scenario B mobile case
8	Scenario B PC case	Scenario B mobile case	Scenario A PC case	Scenario A mobile case
9	Scenario A PC case	Scenario A mobile case	Scenario B PC case	Scenario B mobile case
10	Scenario B PC case	Scenario B mobile case	Scenario A mobile case	Scenario A PC case
11	Scenario A mobile case	Scenario A PC case	Scenario B mobile case	Scenario B PC case
12	Scenario B PC case	Scenario B mobile case	Scenario A PC case	Scenario A mobile case
13	Scenario A PC case	Scenario A mobile case	Scenario B PC case	Scenario B mobile case
14	Scenario B mobile case	Scenario B PC case	Scenario A mobile case	Scenario A PC case
15	Scenario A mobile case	Scenario A PC case	Scenario B PC case	Scenario B mobile case
16	Scenario B PC case	Scenario B mobile case	Scenario A PC case	Scenario A mobile case
17	Scenario A mobile case	Scenario A PC case	Scenario B mobile case	Scenario B PC case
18	Scenario A PC case	Scenario A mobile case	Scenario B PC case	Scenario B mobile case
19	Scenario B mobile case	Scenario B PC case	Scenario A PC case	Scenario A mobile case
20	Scenario A mobile case	Scenario A PC case	Scenario B mobile case	Scenario B PC case

21	Scenario B mobile case	Scenario B PC case	Scenario A PC case	Scenario A mobile case
22	Scenario A mobile case	Scenario A PC case	Scenario B mobile case	Scenario B PC case
23	Scenario B mobile case	Scenario B PC case	Scenario A mobile case	Scenario A PC case
24	Scenario A PC case	Scenario A mobile case	Scenario B PC case	Scenario B mobile case
25	Scenario B mobile case	Scenario B PC case	Scenario A mobile case	Scenario A PC case
