# Supplementary material for: The Effectiveness of Near-Field Communication Integrated with a Mobile Electronic Medical Record System: Emergency Department Simulation Study
Source: JMIR Mhealth Uhealth. 2018 Sep 21;6(9):e11187. doi: 10.2196/11187 (PMC6231820; doi:10.2196/11187)
Supplement: Multimedia Appendix 2 [file mhealth_v6i9e11187_app2.pdf]

Multimedia Appendix 2.

Task	N (%)	PC-mobile total time interval (seconds), (95% CI)	*P* value
**Scenario A**			
**Age groups (years)**			
≥30	12 (48)	26.7 (18.2-35.6)	.17
<30	13 (52)	43.9 (27.6-74.1)	
**Gender**			
Male	14 (56)	27.1 (21.8-32.4)	.32
Female	11 (44)	46.5 (25.2-78.5)	
**Occupation**			
Resident	19 (76)	37.8 (25.2-57.0)	.64
Specialist	6 (24)	28.7 (16.0-44.7)	
**Scenario B**			
**Age groups (years)**			
≥30 or over	12 (48)	42.0 (36.2-48.9)	.03
<30	13 (52)	31.0 (24.9-37.0)	
**Gender**			
Male	14 (56)	38.6 (34.2-43.8)	.34
Female	11 (44)	33.3 (24.5-43.7)	
**Occupation**			
Resident	19 (76)	32.9 (28.3-37.0)	.01
Specialist	6 (24)	47.0 (36.4-59.0)	
