# Supplementary figures and images for: The Effectiveness of Near-Field Communication Integrated with a Mobile Electronic Medical Record System: Emergency Department Simulation Study
Source: JMIR Mhealth Uhealth. 2018 Sep 21;6(9):e11187. doi: 10.2196/11187 (PMC6231820; doi:10.2196/11187)

## Slide 1
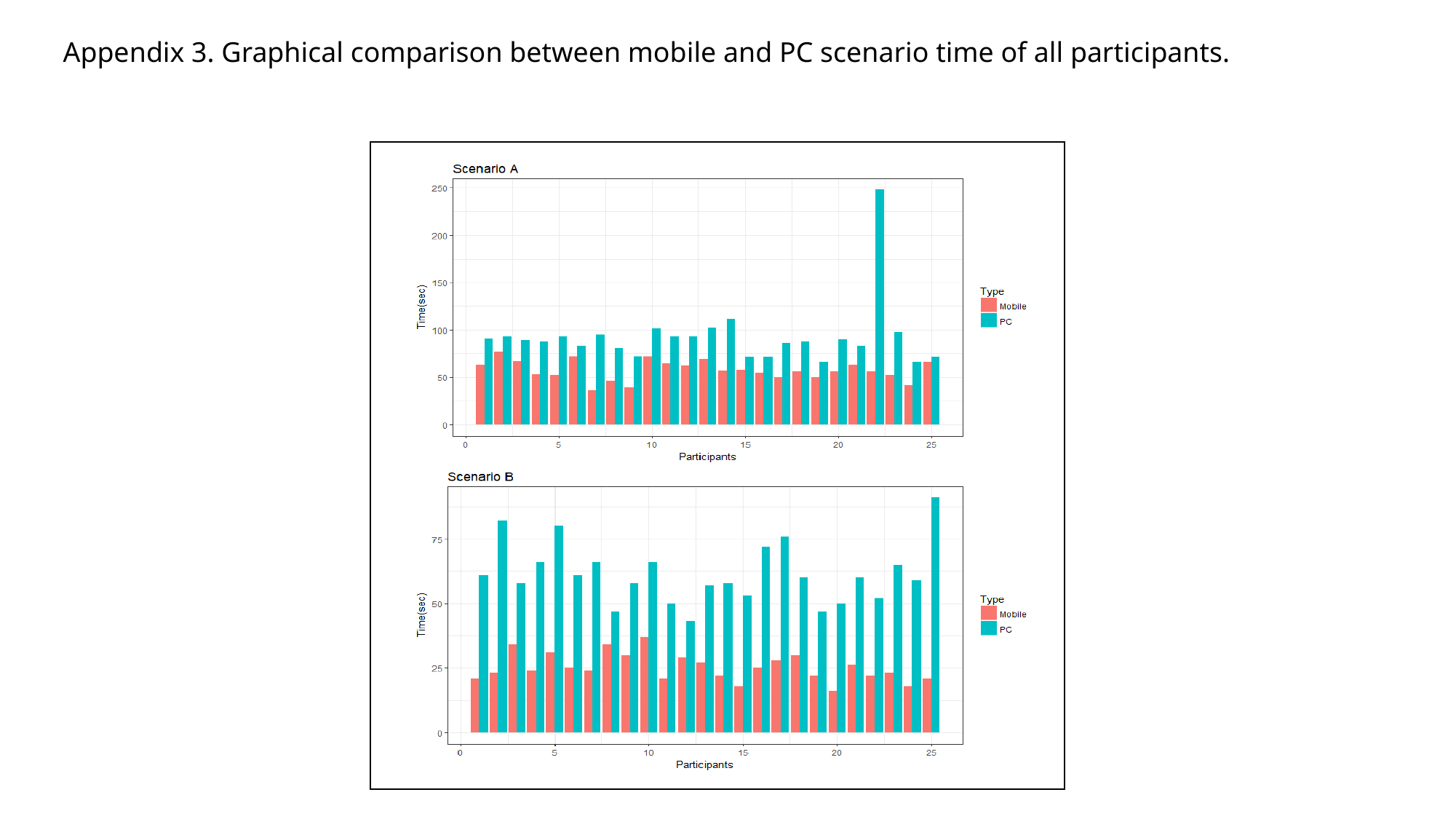

Appendix 3. Graphical comparison between mobile and PC scenario time of all participants.

Supplement: Multimedia Appendix 3 [file mhealth_v6i9e11187_app3.pptx]
